# *Cryptococcus* escapes host immunity: What do we know?

**DOI:** 10.3389/fcimb.2022.1041036

**Published:** 2022-10-13

**Authors:** Chen Yang, Yemei Huang, Yangyu Zhou, Xuelei Zang, Hengyu Deng, Yitong Liu, Dingxia Shen, Xinying Xue

**Affiliations:** ^1^ Department of Laboratory Medicine, the First Medical Centre, Chinese People's Liberation Army (PLA) General Hospital, Beijing, China; ^2^ Department of Respiratory and Critical Care, Beijing Shijitan Hospital, Capital Medical University, Peking University Ninth School of Clinical Medicine, Beijing, China; ^3^ School of Clinical Medicine, Weifang Medical University, Weifang, China

**Keywords:** *Cryptococcus*, *Cryptococcus neoformans*, *Cryptococcus gattii*, immune escape, cellular immunity, humoral immunity, cytokines

## Abstract

*Cryptococcus* is an invasive fungus that seriously endangers human life and health, with a complex and well-established immune-escaping mechanism that interferes with the function of the host immune system. *Cryptococcus* can attenuate the host’s correct recognition of the fungal antigen and escape the immune response mediated by host phagocytes, innate lymphoid cells, T lymphocytes, B lymphocytes with antibodies, and peripheral cytokines. In addition, the capsule, melanin, dormancy, Titan cells, biofilm, and other related structures of *Cryptococcus* are also involved in the process of escaping the host’s immunity, as well as enhancing the ability of *Cryptococcus* to infect the host.

## 1 Introduction

The *Cryptococcus* genus is affiliated with Basidiomycota, Agaricmycotina, Tremelloymcetes, and Tremellales (Heitman et al., 2010), and is a group of capsule-covered opportunistic pathogenic fungi. Of the 37 species known, *Cryptococcus neoformans* (Cn) and *Cryptococcus gattii* (Cg) are the main human pathogenic cryptococci (Franco-Paredes et al., 2015). Cn is widespread in nature, often originating from pigeon feces, and mainly infects immunocompromised individuals, such as the elderly, HIV/AIDS patients, and organ transplant recipients, as well as often causing central nervous system infections (NYu et al., 2021). It is estimated that 223,000 people with HIV/AIDS develop cryptococcal meningitis each year, of which 181,000 die (Rajasingham et al., 2017). Cg was initially thought to be transmitted only in tropical and subtropical eucalyptus areas; there has been a steady increase in the number of Cg infections in the world in temperate regions since the outbreak on Vancouver Island. Cg can infect both immunocompetent and immunosuppressed people, and has a mortality rate of nearly 33% (Jamil et al., 2020). *Cryptococcus* has become a lethal pathogen that poses a serious threat to public health safety (Jamil et al., 2020).

The host immune system has established a large and complex immune mechanism in response to invasion by *Cryptococcus* (reviewed in Hernández-Chávez et al., 2017). Cryptococci invade the host alveoli *via* the respiratory tract in the form of fungi or fungal spores, and are then exposed to the host immune response with phagocytes as the first barrier. The pattern recognition receptors (PRRs) of alveolar phagocytes recognize the conserved pathogen-associated molecular patterns (PAMPs) of *Cryptococcus* and mediate the formation of phagosomes by macrophages, dendritic cells, neutrophils, etc. (Hatinguais et al., 2020). In addition, phagocytes can secrete large amounts of cytokines and chemokines, and can act as antigen-presenting cells (APCs) to present antigens to T lymphocytes for the clearance of cryptococci through cellular and humoral immunity (reviewed in Li et al., 2019).

However, *Cryptococcus* has also evolved a targeted and well-established immune escape capability from being identified and killed (reviewed in Hernández-Chávez et al., 2017). In this review, we focus on and determine the mechanisms of *Cryptococcus* immune escaping, as well as the fungal structure associated with *Cryptococcus* escaping immunity.

## 2 Mechanisms by which *Cryptococcus* escapes host immunity

### 2.1 Immune escape of host cellular immunity

#### 2.1.1 Immune escape of phagocytes

##### 2.1.1.1 Immune escape of macrophages

**Figure 1:**
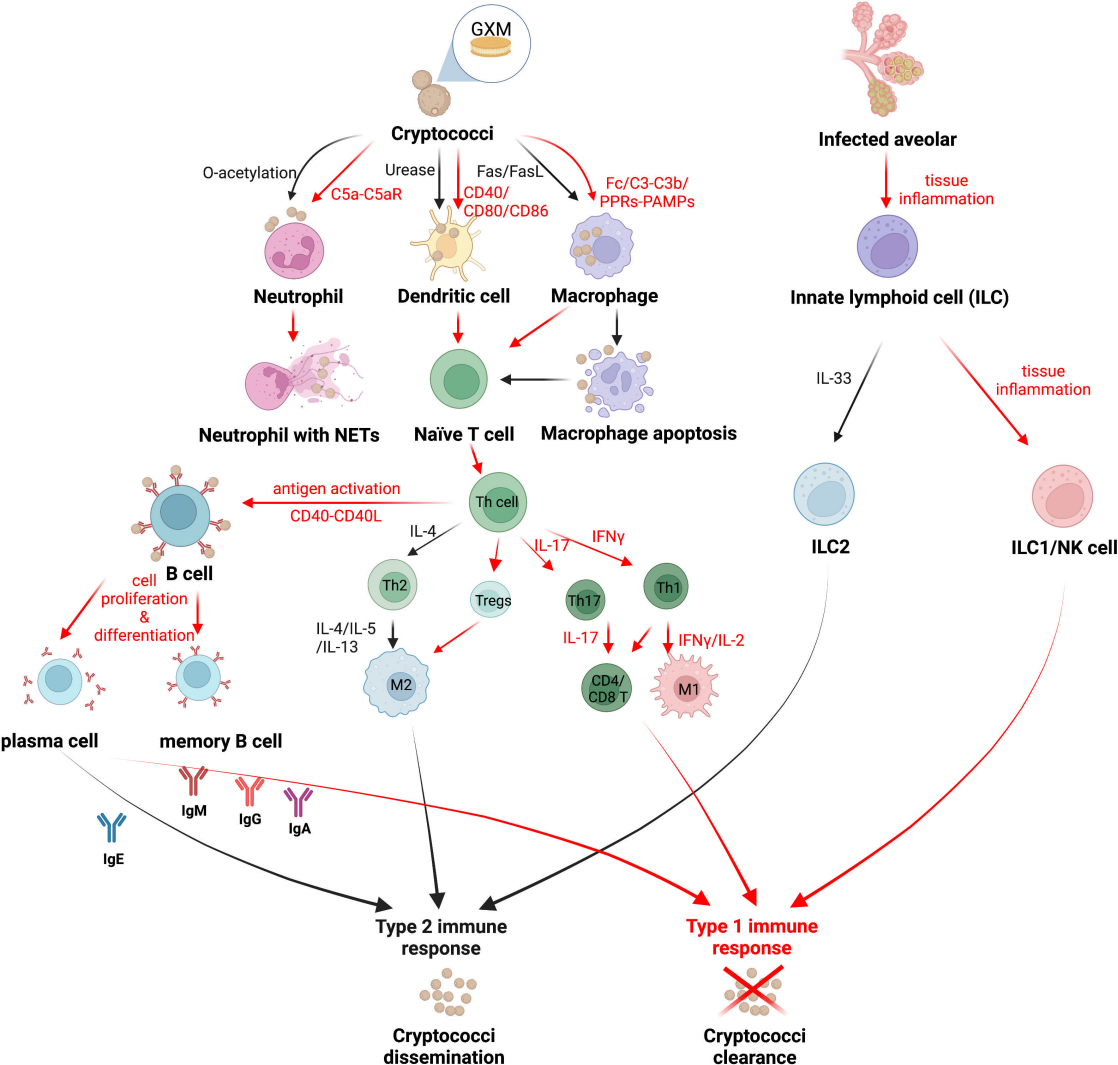
The strategy of *Cryptococcus* immune escaping. *Cryptococcus* can attenuate the host’s correct recognition of the fungal antigen components and escape the immune response mediated by host phagocytes. Red arrows indicate the mechanism by which the host immune system clears *Cryptococcus*, and black arrows indicate the mechanism by which *Cryptococcus* escapes host immunity. Created in BioRender.com.

Macrophages that infiltrate in large numbers into the alveoli are the first line of defense of the host immune system against cryptococci (Trevijano-Contador et al., 2016). The immune process of macrophages against *Cryptococcus* includes capsule polysaccharide recognition, phagocytosis, killing, cytokine and chemokine production, and antigen presentation (Barreto-Bergter and Figueiredo, 2014; Trevijano-Contador et al., 2016). Previous studies have suggested that the phagocytic process of *Cryptococcus* is mostly dependent on opsonin, including antibody and complement opsonization, such as antibody receptor in the Fc region against the capsule antigen component glucuronoxylomannan (GXM) in addition to the capsule-dependent C3-C3b complement system, which together complete the phagocytosis and internalization of *Cryptococcus* (reviewed in Rohatgi and Pirofski, 2015; Sun et al., 2019). However, recent studies have shown that macrophages can also phagocytose pathogens in a non-opsonic manner, i.e., through the direct recognition of fungal cell wall PAMPs by PRRs (Sun et al., 2019). In contrast to the “mannoprotein receptor-mannoprotein” recognition mode of other fungi, macrophages rely on the spleen tyrosine kinase pathway to recognize *Cryptococcus*, in which Cn can be recognized by both Dectin-1 and Dectin-2 receptors, whereas Cg is only recognized by Dectin-1 receptors, and the difference is determined by the different PAMPs in the cell walls of the two species (Lim et al., 2018) (**Figure 1**).

The activation state of macrophages includes the classically activated M1 type, which is protective of the host, and the alternatively activated M2 type, which is nonprotective. M1/M2 macrophages can be interconverted by immediate changes in cytokines in the immune microenvironment (Davis et al., 2013; Subramani et al., 2020). M1 macrophages are activated by IFN-γ produced by type 1 immune responses *via* the signal transduction and activator of transcription-1 (STAT1) signaling pathway, which in turn produces reactive oxygen species (ROS) and reactive nitrogen species (RNS) to kill pathogenic bacteria (Leopold Wager and Wormley, 2014). M2 macrophages, in turn, are activated by IL-4 and IL-13 of type 2 immune responses with the induction of Arg1 and CD206 (mannose receptor) expression (Leopold Wager and Wormley, 2014; Subramani et al., 2020). Furthermore, Müller U (Müller et al., 2012) et al. demonstrated that the interleukin-4 receptor (IL-4R) on T helper (Th) cells suppressed host resistance in pulmonary cryptococcosis with an enhancing type 2 immunity. Cryptococci cannot be eliminated by M2 macrophages; instead, cryptococci can grow, multiply, and spread within M2 macrophages or even go dormant in preparation for latent infection (Subramani et al., 2020). Interestingly, cryptococci were proved to weaken the fungicidal ability of inflammatory monocytes (IM) and aggravate the progression of infection, which is independent of lymphocyte priming, eosinophil recruitment, or downstream M2 macrophage polarization pathways (Heung and Hohl, 2019). *Cryptococcus* can escape from M1 macrophages by inducing the polarization of macrophages towards the M2 phenotype *via* the Hsp 70 homolog Ssa1 (Eastman et al., 2015a). Recent research has revealed that macrophages, while maximizing the killing of *Cryptococcus*, are also “accomplices” in facilitating the immune escape of *Cryptococcus*. Sabiiti W (Sabiiti et al., 2014) et al. found that macrophages provide an ecological niche for *Cryptococcus* to replicate intracellularly, which limits the effect of extracellular antifungal drugs, as well as the fact that *Cryptococcus* crosses the blood-brain barrier to enter the brain *via* macrophages as a Trojan horse transmission (Santiago-Tirado et al., 2017). Interventional reductions in host macrophages have even been shown to reduce the risk of *Cryptococcus* spreading *in vivo* (Charlier et al., 2009).

It has been reported that the phagocytosed cryptococcal capsule component GXM may induce the apoptosis of macrophages mediated by the Fas/FasL pathway and inhibite subsequent immune activation (Villena et al., 2008). At the same time, *Cryptococcus* has evolved a way of escaping macrophages *via* vesicle encapsulation, known as nonlytic exocytosis, or “vomocytosis”, which not only assists *Cryptococcus* in invading the central nervous system but also protects *Cryptococcus* from the immune system during the incubation period (Santiago-Tirado et al., 2017).

##### 2.1.1.2 Immune escape of dendritic cells

DCs are densely distributed in the host’s respiratory mucosa and serve as a bridge between innate and adaptive immune responses, with their main function being the uptake and presentation of pathogenic antigens (Trombetta and Mellman, 2005; reviewed in Mukaremera and Nielsen, 2017). Similar to macrophages, opsonization by antibodies or complements in the immune environment enhances phagocytosis and the killing of cryptococci by DCs (Campuzano and Wormley, 2018). DCs are induced to mature by costimulatory molecules (CD40, CD80, and CD86, etc.) and migrate to T-cell-rich lymphoid organs, where the major histocompatibility complex class II (MHC-II) on their surface presents the processed antigenic component to the initial T cells, namely mannoprotein (MP), initiating the subsequent T lymphocyte immune response. DCs engulf cryptococci and recruit lysosomes to fuse with them, which are independently segregated within the phagocytosed lysosomes and killed by oxidative and nonoxidative mechanisms, while cathepsin B forms pores in the cryptococci cell wall and accelerates cryptococci lysis (Nelson et al., 2021).

As a heterogeneous population, DCs comprise different subtypes, such as CD11b+ myeloid DCs, plasmacytoid DCs (pDCs), and CD103+ DCs (Condon et al., 2011; Nelson et al., 2020). During cryptococcal infection, CD11b+ DCs migrate to the lung and present antigens to cryptococcal-specific T cells, which is of significance for the clearance of cryptococci (Wozniak et al., 2006; Osterholzer et al., 2009a). The recognition and uptake of *Cryptococcus* by pDCs rely on the expressive level of Dectin-3 and the chemokine receptor CXCR3, and the fungicidal activity of pDCs depends on the production of ROS within the lysosome (Hole et al., 2016). CD103+ DCs infiltrate into the lung mucosa and distribute along the lung vascular wall; however, the CD103+ DCs population occupied a very small proportion of the total DCs (Eastman et al., 2015b; Nelson et al., 2020). Studies on CD103+ DCs in response to pulmonary cryptococcosis deserve more attention (Nelson et al., 2020).

Activated by IL-12, a protective Th1-type cytokine produced by mature DCs in the presence of IFN-γ, Th1 cells possess a positive interrelationship with DCs, together activate the inflammatory response against *Cryptococcus* and enhance host protection (Vieira et al., 2000; reviewed in Mukaremera and Nielsen, 2017), whereas the nonprotective DCs expressing the costimulatory molecules CD86 and OX40L induced Th2 cells to secrete IL-4, IL-5, and IL-13, and recruit eosinophils to participate in the anti-inflammatory response, enhancing the immune escape of *Cryptococcus* from the host (Ohshima et al., 1998; reviewed in Mukaremera and Nielsen, 2017). Cryptococci were found to evade immune surveillance by DCs through the virulence factor urease, which impedes the maturation of DCs and inhibits their antigen presentation role (Osterholzer et al., 2009b). While the *O*-acetyl groups in the GXM of the capsule assist DCs in recognizing cryptococci, the deacetylation of an *O*-acetyl group in the GXM structure of Cg further limits the immune recognition function of DCs compared to Cn, making Cg more immune-evasive from host clearance (Urai et al., 2016) (**Figure 1**).

In addition, Cg phagocytosed by DCs forms a persistent actin cage at the periphery of the phagosome, a cage-like structure that spatially and functionally effectively prevents the fusion of phagosomes with lysosomes as well as inhibiting the formation of phagolysosomes and the further immune activation of DCs, leading to the immune paralysis of Cg by DCs. This unique immune evasion mechanism may be an important reason for the highly virulent phenotype of Cg, and partially explains its ease of infection of immunocompetent hosts (Jamil et al., 2020).

##### 2.1.1.3 Immune escape of neutrophils

Neutrophils are one of the key immune mechanisms for host resistance to cryptococcal infection, and can be rapidly recruited to the site of infection in response to chemokines released by the pathogen or host cells. The augmentation of neutrophil fungicidal activity by stimulation of granulocyte colony stimulating factor (G-CSF) and granulocyte-macrophage colony-stimulating factor (GM-CSF) remarkably reduced the fungal burden and prolonged the survival time (Chiller et al., 2002). The recruitment of neutrophils to *Cryptococcus* requires the activation of the complement C5a-C5aR pathway, as well as the activation of the mitogen-activated protein kinase (MAPK) system of extracellular signal-regulated kinases (ERK) and p38, resulting in the production of large amounts of proinflammatory cytokines, including high concentrations of IL-4, IL-10, IL-12, and TNF-α (Sun et al., 2015). Leukotriene B4 (LTB4), a kind of eicosanoid produced by the expression of complement C3 and CD11b, was quickly released by neutrophils during interactions with cryptococci, motivating large amounts of neutrophils to the site of infection, swarming and engulfing cryptococci (Sun and Shi, 2016). Neutrophils also form neutrophil extracellular traps (NETs) to trap cryptococci, and NETs limit the retaining and killing of cryptococci just within their own traps, so as to prevent excessive inflammatory factors associated with these traps from causing damage to the body (Springer et al., 2010; reviewed in Hernández-Chávez et al., 2017) (**Figure 1**).

However, it was found that although cryptococcal capsule GXM has chemotactic activity against neutrophils, the *O*-acetyl groups in it inhibit neutrophil migration and phagocytosis in addition to even restricting the formation of NETs, enhancing the ability of *Cryptococcus* to escape the immune effects of the host (Rocha et al., 2015; reviewed in Campuzano and Wormley, 2018). In addition, GXM induces the shedding of L-selectin and TNF-α receptors on the surface of neutrophils, thereby reducing neutrophil adhesion to the endothelial surface of blood vessels and preventing their migration to the site of infection, which explains the reduced infiltration of neutrophils into host tissues infected with disseminated cryptococci (Ellerbroek et al., 2004).

#### 2.1.2 Immune escape of innate lymphoid cells

Innate lymphoid cells (ILCs), a vital part of the innate immune system, are stemmed from lymphoid lineages lacking PRRs (Spits et al., 2013). Although ILCs fail to recognize antigens specifically, they contribute immediately in tissue homeostasis, pathological inflammation, and immunity against infections through cytokine stimulation bringing about direct subsequent innate and adaptive immune responses (Elemam et al., 2021). ILCs are generally classified into three major subgroups of innate lymphoid cells: group 1 (ILC1s), including natural killer (NK) cells, group 2 (ILC2s), and group 3 (ILC3s) (Spits et al., 2013). ILC1s drive type 1 immune responses which respond to intracellular pathogenic bacteria and fungi with the secretion of IFN-γ and TNF (Elemam et al., 2021). NK cells play crucial roles in fungi clearance in various ways, including cytokine production (IFN-γ, GM-CSF, and TNF-α) and the secretion of cytotoxic molecules secretion (perforin, granzymes, and granulysin) (Elemam et al., 2021). Granulysin participates in interference with oxidative metabolism and the energy generation of fungi, and it can also directly kill extracellular pathogens by altering the membrane integrity, while further decreasing the viability of intracellular pathogens when combined with perforin (Elemam et al., 2021). ILC2s mediate type 2 immune responses producing Th2 cytokines, including IL-4, IL-5, and IL-13 (Elemam et al., 2021). ILC3s are found to act as equivalents of Th17 cells, and mainly maintain the microbiota-host homeostasis of intestinal immunity (Elemam et al., 2021).

Recent studies have illustrated that the activation of ILC1s and M1-type macrophages strongly restricts Cn infections during cryptococcosis, whereas type 2 immunity plays a reverse role in disease progression that exacerbates pulmonary infections with ILC2s as well as M2-type macrophages (Kindermann et al., 2020). Activated by the stimulation of alarmin IL-33, ILC2s were shown to exert high amounts of type 2 cytokines, which modulate mucus hyperproduction, eosinophilia, and the activation of alternatively activated macrophages (M2) in infected organs (Kabata et al., 2018; Kindermann et al., 2018). Furthermore, it was demonstrated that highly virulent *Cryptococcus* strains induced type 2 immunity significantly, including ILC2s induction, by triggering the alarmin IL-33 in alveolar type 2 epithelial cells (Flaczyk et al., 2013; Heyen et al., 2016; Knipfer et al., 2019; Kindermann et al., 2020). Remarkably, ILC2s suppress pulmonary type 1 immunity and classical macrophage (M1) activation, resulting in attenuated fungal control, suggesting a detrimental role of ILC2s during cryptococcosis (Kindermann et al., 2020) (**Figure 1**).

#### 2.1.3 Immune escape of T lymphocyte

##### 2.1.3.1 Immune escape of effector T lymphocytes

T-lymphocyte-mediated immune responses contributed greatly against *Cryptococcus*, as phagocytes process cryptococcal antigens and present them to T lymphocytes, inducing the initial T cells to proliferate and mature as well as differentiate into various subtypes (Elsegeiny et al., 2018). Studies have shown that both effector CD4+ T cells and CD8+ T cells are involved in the immune response to *Cryptococcus*, and both proliferate in large numbers as well as produce granulysin to kill cryptococci (Zheng et al., 2007). Pulmonary infiltrating effector T lymphocytes stimulated by cryptococcal antigens secrete both protective Th1-type (IFN-γ, IL-2) and Th17-type (IL-17) cytokines, as well as nonprotective Th2-type (IL-4, IL-5) cytokines (reviewed in Mukaremera and Nielsen, 2017; Mills, 2022). Both CD4+ T and CD8+ T cells produce anticryptococcal Th1-type and Th17-type cytokines, whereas Th2-type cytokines are only produced by CD4+ T cells (Mills, 2022). It was found that *Cryptococcus* was not sitting still and that capsule GXM prompted IL-4 and IL-10 secretion while inhibiting TNF-α and IFN-γ production, enhancing the ability of *Cryptococcus* to escape the killing effects of CD4+ T and CD8+ T cells (Scriven et al., 2016) (**Figure 1**). IL-4R was proven to be a major determinant affecting the host susceptibility to cryptococcosis, which is expressed on Th cells *via* Th2 responses (Müller et al., 2012). IL-4R complexes can bind to the IL-4 and IL-13 involved in Th2 responses, suppressing host resistance in pulmonary cryptococcosis with an enhancing type 2 immunity. Conversely, lacking IL-4R on Th cells shows a protective classically activated macrophages (M1 macrophages) response and controls the pulmonary cryptococcosis (Müller et al., 2012).

In addition, immunocompetent CD4+ T cells also cause the host to develop immune reconstitution inflammatory syndrome (IRIS) and postinfectious inflammatory response syndrome (PIIRS), which severely disrupt the central nervous system (CNS) (Shourian and Qureshi, 2019). Neal et al. (2017) found that although CD4+ T cells significantly reduced the fungal load of target organs, more than half of the mice died from neurological dysfunction in the late stages of infection. This explains in part the high morbidity and mortality associated with CNS cryptococcal infection in immunocompetent patients. *Cryptococcus* causes an immune overreaction in the host that disrupts the immune homeostasis and triggers a lethal inflammatory response.

##### 2.1.3.2 Immune escape of other subtypes of T lymphocytes

Regulatory T cells (Tregs) are one of the CD4+ T cell subtypes that negatively regulate the host immune response during most fungal infections (Wiesner et al., 2016). When infected with *Histoplasma* and *Candida albicans*, fungal clearance is promoted with increasing proinflammatory cytokines and a reduction in Tregs. However, Tregs were discovered to suppress the nonprotective Th2 immune response during infections with *Pneumocystis* and *Cryptococcus*. With the assistance of the C-C chemokine receptor type 5 (CCR5) and IFN regulatory factor 4 (IRF4) in cryptococci-infected lungs, Tregs are able to localize the infected sites and subsequently suppress Th2 effector cells subsequently (Schulze et al., 2016; Wiesner et al., 2016). During pulmonary cryptococcal infection, elevating Tregs lead to limit *Cryptococcus*-infection-induced allergic airway inflammation by reducing IgE, mucus production, and Th2 cytokine production, while not altering the cryptococci burden (reviewed in Schulze et al., 2016; Mukaremera and Nielsen, 2017) (**Figure 1**).

Natural killer T (NKT) cells, especially Vα14+ NKT cells, activated by cryptococcal lipid antigens presentation *via* DCs and recruited to the lung by monocyte chemoattractant protein-1 (MCP-1) chemokines (Kawakami et al., 2001), play an important role in inducing a protective Th1 immune response. Further studies on Vα14+ NKT cells have revealed that, when activated by α-galactosylceramide, Vα14+ NKT cells induced Th1 immune responses with IFN-γ synthesis during *Cryptococcus* infection. At the same time, NKT cells also cause delayed-type hypersensitivity in the host and a local allergic inflammation in the lung, even necrosis (Sato et al., 2020).

γδ T cells are also activated by cryptococcal antigens presented by macrophages and DCs, while MCP-1 is not required during the accumulation. Notably, γδ T cells inhibit IFN-γ synthesis and downregulate protective Th1 immune responses, which impede host immunity towards cryptococci (Wiesner et al., 2016; reviewed in Mukaremera and Nielsen, 2017). The various subtypes of T lymphocytes in the host are functionally interlocked, allowing the opportunity for *Cryptococcus* to escape host immune clearance.

### 2.2 Immune escape of host humoral immunity

The humoral immune responses against cryptococci are mainly mediated by B lymphocytes and antibodies. B-1 cells, a subtype of mature B cells, play a crucial role in enhancing resistance to and preventing the dissemination of cryptococci. Antibodies produced against cryptococcal proteins and capsule GXM are detected in the sera of the vast majority of children, suggesting that humans can develop humoral immune responses to environmental exposure to cryptococci at an early age (Goldman et al., 2001). B-1 cells from hosts infected with *Cryptococcus* secrete cryptococci-binding IgM, the depletion of which would result in reduced alveolar macrophages phagocytosis and increased fungal dissemination to the brain (Rohatgi and Pirofski, 2012). IgM suppresses pulmonary cryptococcal load and reduces host susceptibility to cryptococci, as well as limiting cryptococcal Titan cell formation (García-Barbazán et al., 2016; Trevijano-Contador et al., 2020), while IgG antibodies enhance the clearance of cryptococci by splenic NK cells (Nabavi and Murphy, 1986; reviewed in Mukaremera and Nielsen, 2017). The levels of serum GXM-binding IgM and IgG are far lower in HIV-infected than HIV-uninfected individuals, as well as in HIV-uninfected organ transplant recipients infected with *Cryptococcus* than those who are not (Jalali et al., 2006). It has also been shown that B lymphocytes and antibodies prevent systemic infection with *Cryptococcus* when T lymphocyte immune function is impaired (Aguirre and Johnson, 1997; reviewed in Mukaremera and Nielsen, 2017).

Moreover, the antibodies also act as opsonins to enhance the phagocytic uptake of cryptococci (Aslanyan et al., 2017). Anti-β-glucan monoclonal antibodies and anti-GXM monoclonal antibodies, which bind to the cryptococcal cell wall and capsule separately, enhance the macrophage killing of cryptococci *in vitro* (Torosantucci et al., 2009; Probert et al., 2019). The prophylactic infusion of anti-*Cryptococcus* antibodies can also control the fungal load of target organs and reduce the damage caused by cryptococcal infection (Torosantucci et al., 2009).

In a previous review, antibodies such as IgM, IgG, and IgA were attributed to protect hosts against *Cryptococcus* (reviewed in Mukaremera and Nielsen, 2017). However, diverse studies have revealed that the protective efficacy of antibodies against cryptococci is dependent on not only antibodies’ classification, but also their specificity. Two clonally related immunoglobulin M (IgM) monoclonal antibodies (MAbs) (12A1 and 13F1) differ in specificity and protective efficacy, presumably due to variable (V)-region sequence differences. MAb 12A1 is protective in *Cryptococcus* infection, while MAb 13F1 is nonprotective (Nakouzi et al., 2001). As for IgGs sharing identical V regions, IgG1, IgG2a, and IgG2b MAbs to the capsular glucuronoxylomannan of *Cryptococcus* prolong the host survival, while IgG3 displays a nonprotective role in enhancing infection (Beenhouwer et al., 2001). IgA mediates the complement-independent phagocytosis of *Cryptococcus* by macrophages with the assistance of complement receptor 3 (CR3) expression, promoting protective efficacy in hosts (Taborda and Casadevall, 2002). Furthermore, increased IgE is associated with a nonprotective type 2 immune response that exacerbates the *Cryptococcus* infection (Zaragoza et al., 2007; Qiu et al., 2013) (**Figure 1**). These results suggest that antibody-mediated immunity against cryptococcal infection is a complex process and that cryptococci can survive within the host humoral immune environment through an immune escape effect.

### 2.3 Immune escape of host cytokine-mediated immune responses

Cytokines are small molecular proteins that mediate the interactions and signaling functions between different immune cells. During cryptococcal infection, protective and nonprotective cytokines are produced by the host.The main protective cytokines include IFN-γ, IL-12, and IL-2, all of which enhance the macrophage expression of ROS in addition to RNS phenotypes and reduce the target organ fungal load, while decreasing host susceptibility (Wang et al., 2015; Firacative et al., 2018). In addition, a class of supporting cytokines are involved in the induction of Th1-type immune responses, including TNF-α, IL-6, IL-8, IL-18, IL-23, and IP10. Of these, TNF-α, IL-6, IL-8 and IP10 are associated with prognoses in patients with HIV/AIDS cryptococcal meningitis (Jarvis et al., 2015; Mora et al., 2015) and IL-6 is also involved in anticryptococcal drug resistance (Blasi et al., 1995).

Nonprotective cytokines are usually produced by Th2-type immune responses, mainly IL-4, IL-5, and IL-13, all of which induce increased lung fungal load and lung eosinophil production, as well as enhanced host susceptibility (Wiesner et al., 2015; Liu et al., 2020). In addition, high levels of IL-13 in cerebrospinal fluid are positively associated with high morbidity and mortality in patients infected with HIV/AIDS cryptococcal meningitis (Scriven et al., 2015). Th1-type and Th2-type immune responses are supposed to be some of the mechanisms by which the body maintains immune homeostasis, and cryptococci break this homeostasis by suppressing Th1-type and enhancing Th2-type immune responses, escaping host immune surveillance and killing.

In addition, there is a specific nonprotective cytokine-related immune response known as immune reconstitution inflammatory syndrome (IRIS), a pathological inflammation that results from an over-recovery of the body’s immune response following antiretroviral therapy in patients with HIV/AIDS cryptococcal meningitis (Eschke et al., 2015). Studies have confirmed that multiple cytokines are associated with cryptococcal IRIS, including serum proinflammatory cytokines such as TNF-α, granulocyte colony-stimulating factor (G-CSF), granulocyte-macrophage colony-stimulating factor (GM-CSF) and vascular endothelial growth factor (VEGF) (Brienze et al., 2021) (**Table 1**).

**Table 1:** Cytokine function based on the interaction between *Cryptococcus* and host immunity.

Classification	Cytokines	Function	References
Protective cytokines	IFN-γ IL-12 IL-2	Th1-type cytokines. Decrease lung and brain fungal burden, lung eosinophilia, fungal dissemination to the brain and susceptibility to infection. Induce numbers of macrophages expressing inducible nitric oxide synthase.	(Wang et al., 2015; Mukaremera and Nielsen, 2017; Firacative et al., 2018)
Protection support cytokines	TNF-α IL-6 IL-8 IL-18 IL-23 IP10	Induce or promote the three major protective cytokines (IFN-γ, IL-12 and IL-2). TNFα, IL-8, IL-6 and IP10 was associated with improved outcome in AIDS patients with CM. IL-23 and IL-18 play a protective role against cryptococcal IL-23 and IL-18 play a protective role against cryptococcal infection.	(Jarvis et al., 2015; Mora et al., 2015; Mukaremera and Nielsen, 2017)
Non-protective cytokines	IL-4 IL-5 IL-13	Th2-type cytokines. Increase lung fungal burden, pulmonary eosinophilia, sensitivity to *Cryptococcus* infection.	(Scriven et al., 2015; Wiesner et al., 2015; Mukaremera and Nielsen, 2017; Liu et al., 2020)
IRIS-related cytokines	TNF-α CSF GM-CSF VEGF	The absence/reduction of serum TNFα, G-CSF, GM-CSF and VEGF predispose AIDS patient with CM to developing subsequent CM-IRIS.	(Eschke et al., 2015; Mukaremera and Nielsen, 2017; Brienze et al., 2021)

AIDS, acquired immune deficiency syndrome; CM, cryptococcal meningitis; IRIS, immune reconstitution inflammatory syndrome; G-CSF, granulocyte-colony-stimulating factor; GM-CSF, granulocyte-macrophage colony-stimulating factor; VEGF, vascular endothelial growth factor. G-CSF, granulocyte-colony-stimulating factor; GM-CSF, granulocyte-macrophage colony-stimulating factor; VEGF, vascular endothelial growth factor.

## 3 Fungal structure and corresponding immune escape mechanisms associated with *Cryptococcus* escaping host immunity

### 3.1 Cryptococcal fungal structure and immune escape

#### 3.1.1 Immune escape effect of the capsule

**Figure 2:**
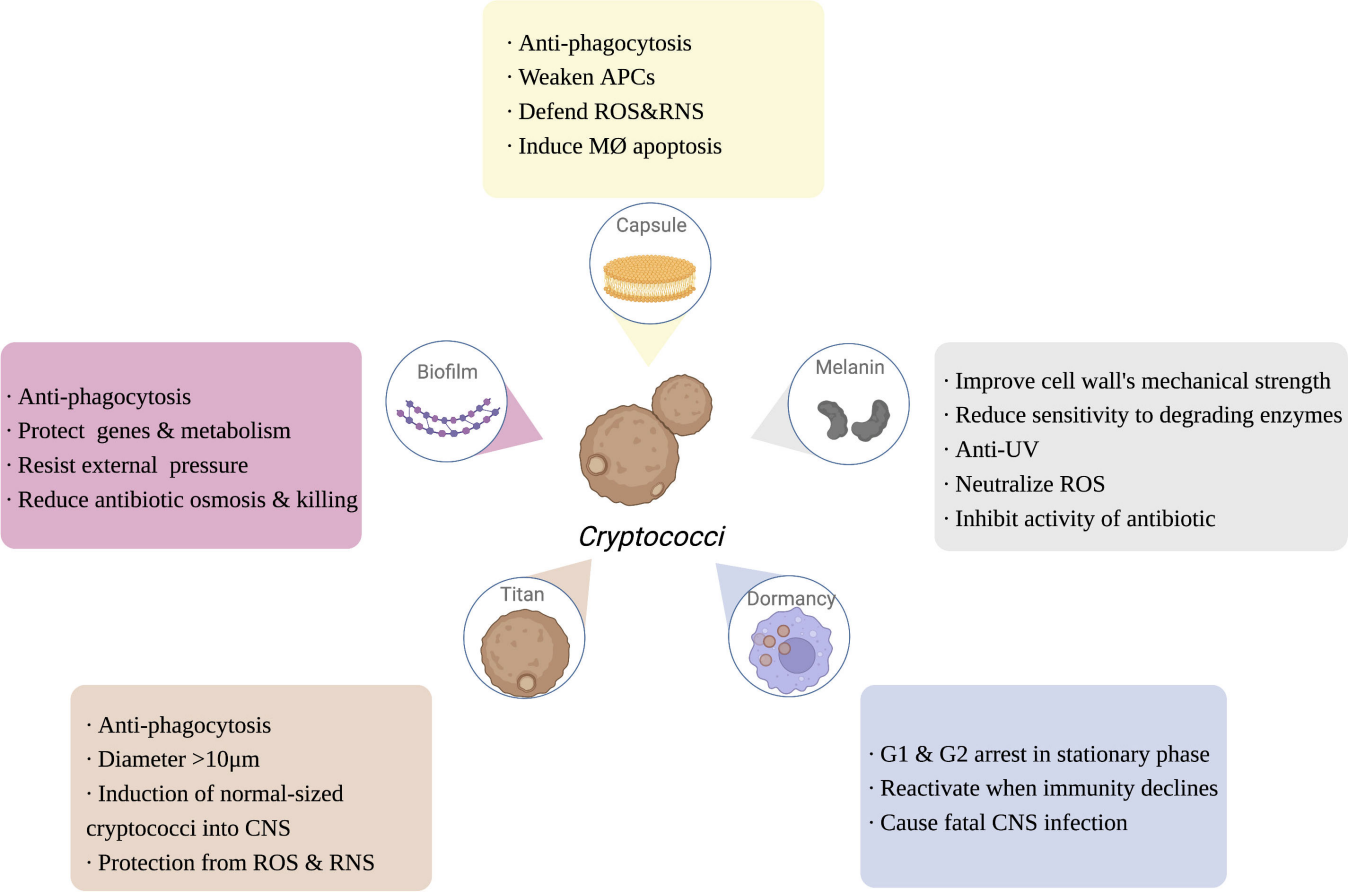
The function of *Cryptococcus* immune escaping. Capsule, melanin, dormancy, titan cells and biofilm of *Cryptococcus* are also involved in the process of escaping the host’s immunity, enhancing the ability of *Cryptococcus* to infect the host. APCs, antigen presenting cells; ROS, reactive oxygen species; RNS, reactive nitrogen species; MØ, APCs, antigen presenting cells; ROS, reactive oxygen species; RNS, reactive nitrogen species; MØ, macrophage; UV, ultra violet; CNS, central nervous system. Created in BioRender.com.

Polysaccharide capsules (PC) are the outermost protective structure of *Cryptococcus*, activating and participating throughout the host immune response, including phagocytic uptake, antigen presentation, antibody production, and mediating the production of functionally diverse cytokines (O'Meara and Alspaugh, 2012; reviewed in Rohatgi and Pirofski, 2015). PC is composed of 90%-95% GXM, 5% galactoxylomannan (GalXM), and less than 1% mannoprotein (Reuwsaat et al., 2018). As the most important virulence factor of *Cryptococcus*, the capsule is also the most important immune-escape-related structure of *Cryptococcus*. In the host, the capsule has multiple self-protective functions, including inhibiting macrophage phagocytosis, reducing the antigen presentation capacity of APCs, downregulating inflammatory cytokine levels, and depleting complement components, thereby attenuating the host immune response (O'Meara and Alspaugh, 2012; Denham et al., 2018; Colombo et al., 2019; Freitas and Santos, 2021). Additionally, once in macrophages, the capsule can also resist killing by host ROS and RNS (Naslund et al., 1995; Casadevall et al., 2019). The capsule can actively sense the immune environment, induce Titan cell formation, and even drive the fungi into dormancy, and its major component, GXM, can induce the apoptosis of macrophages mediated by the Fas/FasL pathway, inhibiting subsequent activation of immune function in addition to maximizing the protection of *Cryptococcus* from attack and clearance by the host immune system (Santiago-Tirado et al., 2017). Moreover, GalXM contributes to immune escaping by inducing apoptosis of T cells, which attributes to the caspase 8 activation and the consequent DNA fragmentation (Pericolini et al., 2006). Denham (Denham et al., 2018) compared the virulence and immune escape of the cryptococcal capsule to “a sword and a shield”, illustrating its crucial function (**Figure 2**).

The analysis of inositol polyphosphate kinases (IPKs) in Cn by Li et al. (2017) revealed that, among IPKs, cell-wall-related Arg1 regulates capsule production to defend phagocytosis by the innate immune system. Greater exposure of surface β-glucan and mannoprotein by reduced capsule size in the *arg1*Δ cryptococci mutant to the binding of opsonizing antibodies is suggested to increase phagocyte recognition and phagocytosis. Furthermore, the polysaccharide capsule of Cg was demonstrated to block the functional maturation of human-monocyte-derived DCs, thereby reducing subsequent *Cryptococcus* phagocytosis (Huston et al., 2016). It was also found that GXM not only adheres to the surface of the cryptococcal cell wall to form capsules, but also enters the blood or cerebrospinal fluid in the free form of exo-GXM. Exo-GXM inhibits the migration of immune cells to the brain and also suppresses the host systemic inflammatory response, thereby exacerbating the host systemic infection, especially in the central nervous system (Denham et al., 2018).

#### 3.1.2 Immune escape effect of melanin

Melanin is a negatively charged, high-molecular-weight hydrophobic substance within the cell wall of *Cryptococcus*, which helps to improve the mechanical strength of the cell wall, enhance resistance to environmental UV light, and reduce the sensitivity of the fungus to degradative enzymes, while melanin reduces cytokine reactivity, attenuates phagocytosis, neutralizes the oxidative substances (e.g., ROS) released by inflammatory cells, and inhibits antifungal drugs activity, serving to protect the fungus and escape from host immune function (reviewed in Nosanchuk et al., 2015; Eisenman et al., 2020). Melanin is regulated by a synthetic network of core transcription factors, including Bzp4, Usv101, Hob1, and Mbs1, as well as the core kinases Gsk3 and Kic1, which all provide possible targets for the clinical development of drugs that inhibit melanin synthesis (Lee et al., 2019a) (**Figure 2**).

### 3.2 Three specific mechanisms of cryptococcal escape immunity

#### 3.2.1 The role of dormancy in immune escape

The close interaction of *Cryptococcus* and phagocytes in the host dictates why it is defined as a facultative intracellular pathogen (Wang et al., 2022). Phagocytes provide a protective niche for *Cryptococcus* to replicate, proliferate, and even be dormant when the host immunity is overexpressed. The ability of *Cryptococcus* to escape the immune effects of the host is enhanced by its greater adaptability to the immune environment (Sorrell et al., 2016). Previous research has observed differences in the cell wall immune recognition epitopes between Cg and Cn, and phagocytic cells were less likely to recognize Cg (Huston et al., 2016), so this section only focuses on the dormant characteristic of Cn to escape host immunity.

Cn infection consists of three stages: early exposure to the immune environment activates the host immune system; subsequently, cryptococci is dormant and latent; and, finally, cryptococci will be reactivated when the immune system declines. Cn is largely nonpathogenic in immunocompetent hosts, and patients are often diagnosed by a pathological biopsy of unexplained pulmonary nodules (Walsh et al., 2019; Alanio, 2020). Studies have shown that the G1 and G2 phases of the interphase of dormant Cn are at standstill, and that highly energetic behaviors such as division and proliferation are terminated, leaving only basic metabolic functions, such as membrane potential and cell morphology, to be maintained (Takeo et al., 1995). However, when the host is exposed to severe immunosuppressive events, such as HIV/AIDS infection, organ transplantation, and severe burns, the dormant cryptococci rapidly activate and cause fatal central nervous system infections, becoming the “last straw” to crush the immunosuppressed patient (Gerstein et al., 2019). Thus, dormancy is essential for the immune escape of Cn, making it the first fungal pathogen to be studied concerning host immunity (Alanio, 2020) (**Figure 2**).

#### 3.2.2 The role of Titan cells in immune escape

Titan cells are macrophage-induced cryptococci larger than 10 μm in diameter, up to 100 μm, which cannot be cleared by alveolar macrophages and are an important immune escape mechanism for *Cryptococcus* (Crabtree et al., 2012; Dambuza et al., 2018). Morphological transition of cryptococci from normal size to titanization was generally stimulated by exposure to external signals, such as CO_2_, hypoxia, and serum stimulation (Dambuza et al., 2018; Trevijano-Contador et al., 2018). Titanization was proved to be related with cAMP-mediated signaling, which depends on Gpr4/Gpr5 receptors, adenylyl cyclase Cac1, Pka1 kinase, and the transcription factor Rim101 (Okagaki et al., 2011; Choi et al., 2012). Electron microscopy revealed that Cryptococcal Titan cells were enlarged (> 10 μm) and contained features such as mononuclear polyploids, large cytoplasmic vesicles, dense capsules, and a broad cell wall (Hommel et al., 2018). Although Titan cells are unable to cause CNS infection *via* the Trojan horse model, it has been found that they can induce a normal size of surrounding cryptococci into the CNS (Crabtree et al., 2012). Recent studies have shown that the transcription factor Pdr802 is involved in regulating the formation of Cryptococcal Titan cells and mediates the entry of *Cryptococcus* into the CNS to cause infection (Reuwsaat et al., 2021). Progeny proliferated by Cryptococcal Titan cells are resistant to unfavorable immune environments such as oxidation and nitrosylation, and have a greater capacity to proliferate within phagocytes (García-Rodas et al., 2019). Titan cells are an important form of *Cryptococcus* resistance to host immune phagocytosis, escape from host immune responses, and exacerbate the infection of the host CNS (**Figure 2**).

#### 3.2.3 The role of biofilms in immune escape

Biofilms are functional three-dimensional structures commonly found on the surface of pathogens that enhance the ability of the organism to survive in a hostile immune environment by protecting important intracellular genetic material and energy metabolic activities against external physical and chemical stresses (Butassi et al., 2021). Biofilm structures can be observed around cryptococci in the host and help *Cryptococcus* to resist the immune effects of the host (Schlafer et al., 2018). Biofilm formation is also observed around cryptococci during nonlytic exocytosis from macrophages, suggesting that biofilms are involved in the transmission of *Cryptococcus* (Lee et al., 2019b). The protective effect of biofilms greatly limits the penetration of antifungal drugs, and therefore cryptococci are often clinically resistant to amphotericin B, fluconazole and voriconazole (Tits et al., 2020) (**Figure 2**). As previous studies have reviewed (reviewed in Moranova et al., 2009; Hernández-Chávez et al., 2017; Alanio, 2020; Rizzo et al., 2021), the strategy of a low proliferation rate and a specific transcriptome of *Cryptococcus* under hypoxia may induce cryptococcal dormancy as well as biofilm formation for immune escaping and survival in host tissue, possibly resulting in a life-threatening secondary acute infection.

## 4 Conclusion

*Cryptococcus* is a serious threat to human life and health; although host immunity plays a major role in defending against *Cryptococcus*, this fatal fungal pathogen has evolved complicated immune escaping strategies to evade surveillance and clearance by the host immune system, enhancing the ability of *Cryptococcus* to infect its hosts. Understanding its immune escaping strategies is crucial for infection control and the development of immunotherapies. This review summarized a series of immune escaping mechanisms of *Cryptococcus*, focusing on anticellular immunity, antihumoral immunity, as well as fungal structures and special mechanisms that contribute to immune escaping. Among these, we have particularly highlighted throughout the detrimental role of the cryptococcal capsule against host immunity. Recent studies (Freitas and Santos, 2021; Rizzo et al., 2021) have revealed that capsular polysaccharide (CP) GXM as well as its extracellular vesicles were deemed as vital targets in the field of developing vaccines against cryptococcosis. Further research on the role of capsule immune escape indeed helps increase the understanding of cryptococcal infection and immune escape, and also sheds new light on enhancing the clearance of cryptococci and solving difficult clinical problems, such as cryptococcal drug resistance.

In addition, normal immunity is an important safeguard against cryptococcal infection but an overactive immune response not only disrupts the body’s immune homeostasis, but also causes an excessive inflammatory response. Therefore, clinical treatment of cryptococcal infections should not only activate the host’s immune function or reduce the immune escape of cryptococci, but also focus on maintaining the body’s immune homeostasis to maximize the patient’s prognosis.

## Author contributions

Writing—original draft preparation, CY; writing—review and editing, YH, YZ, X.Z, HD, YL, DS, XX; figure handling, CY; supervision, DS, XX; funding acquisition, XX. All authors have read and agreed to the published version of the manuscript.

## Funding

This research was funded by the grants from the National Key Research and Development Program (No. 2021YFC2302100), Capital Health Development Scientific Research Unit Matching Fund (No. 2020-2Z-2086), Beijing Hospitals Authority Clinical medicine Development of special funding support (No. XMLX202115), National Capacity Building Project for Multidisciplinary Diagnosis and Treatment of Major Diseases (No. 2019YLFW), Beijing Key clinical specialty project (No. 2020ZDZK1), and National Key Research and Development Program (No. 2020YFC2005404).

## Conflict of interest

The authors declare that the research was conducted in the absence of any commercial or financial relationships that could be construed as a potential conflict of interest.

## Publisher’s note

All claims expressed in this article are solely those of the authors and do not necessarily represent those of their affiliated organizations, or those of the publisher, the editors and the reviewers. Any product that may be evaluated in this article, or claim that may be made by its manufacturer, is not guaranteed or endorsed by the publisher.
